# Carbon monoxide and mitochondria—modulation of cell metabolism, redox response and cell death

**DOI:** 10.3389/fphys.2015.00033

**Published:** 2015-02-09

**Authors:** Ana S. Almeida, Cláudia Figueiredo-Pereira, Helena L. A. Vieira

**Affiliations:** ^1^Chronic Diseases Research Center, NOVA Medical School/Faculdade de Ciências Médicas, Universidade Nova de LisboaLisboa, Portugal; ^2^Instituto de Tecnologia Química e Biológica, Universidade Nova de LisboaOeiras, Portugal; ^3^Instituto de Biologia Experimental e TecnológicaOeiras, Portugal

**Keywords:** mitochondria, carbon monoxide, cytochrome *c* oxidase, reactive oxygen species, mitochondrial biogenesis, mitochondrial metabolism, programmed cell death, gasotransmitters

## Abstract

Carbon monoxide (CO) is an endogenously produced gasotransmitter, which is associated with cytoprotection and cellular homeostasis in several distinct cell types and tissues. CO mainly targets mitochondria because: (i) mitochondrial heme-proteins are the main potential candidates for CO to bind, (ii) many CO's biological actions are dependent on mitochondrial ROS signaling and (iii) heme is generated in the mitochondrial compartment. Mitochondria are the key cell energy factory, producing ATP through oxidative phosphorylation and regulating cell metabolism. These organelles are also implicated in many cell signaling pathways and the production of reactive oxygen species (ROS). Finally, mitochondria contain several factors activating programmed cell death pathways, which are released from the mitochondrial inter-membrane space upon mitochondrial membrane permeabilization. Therefore, disclosing CO mode of action at mitochondria opens avenues for deeper understanding CO's biological properties. Herein, it is discussed how CO affects the three main aspects of mitochondrial modulation of cell function: metabolism, redox response and cell death.

## Introduction

Carbon monoxide (CO) is mostly known as a silent-killer due to its great affinity to hemoglobin, which compromises oxygen delivery and promotes high levels of intoxication and death. Nevertheless, in 1949 CO was found to be an endogenous molecule, exhaled by healthy humans (Sjostrand, 1949). Late, Tenhunen and colleagues described heme oxygenase (HO) enzyme, whose activity is degrading heme group, which gives rise to CO, along with bilirubin and free iron (Tenhunen et al., 1968). Nowadays, HO activity is well accepted to present several beneficial biological functions. There are two known isoforms of this enzyme, which can be expressed (isoform HO-1) or activated (isoform HO-2) in response to numerous biological stresses, namely: oxidative stress, hypoxia, hyperoxia, hypothermia, unfolded protein response, inflammation, and ischemia (Ryter, 2006; Gozzelino et al., 2010). Several reasons are stated for HO to be a homeostatic and cytoprotective enzyme. First, HO is crucial for eliminating free heme, which is a potent damaging molecule, due to its free iron that generates hydroxyl radicals through Fenton reaction (Gozzelino et al., 2010). Secondly, bilirubin is rapidly converted into biliverdin, which is a potent anti-oxidant molecule (Dore et al., 1999; Rodella et al., 2006; Ryter, 2006). Finally, CO is involved in several cellular processes, acting as anti-inflammatory, cytoprotective, maintenance of tissue homeostasis and, in some particular cases, anti-proliferative and vasodilator (Bilban et al., 2008; Motterlini and Otterbein, 2010; Queiroga et al., 2014).

For potential clinical applications of CO, the main scientific and technical challenges are the safe and specific manner of delivering CO. Inhalation of CO gas present several limitations: need of hospital environment and devices, risk of high levels of carboxyhemoglobin and tissue/organ unspecific deliver of CO. The development of CO-releasing molecules (CORMs) emerges as a potential solution for CO deliver, as reviewed in Romao et al. (2012). In experimental approaches (rodent *in vivo* or cell culture models), the most studied CORMs are the sodium boranocarbonate water soluble CORM-A1, the metal-carbonyl based CORM-2 and its water soluble related molecule CORM-3 (Boczkowski et al., 2006).

Mitochondria are the main cellular energy generators through oxidative phosphorylation and participate in several signaling cascades. Mitochondria operate in the adaptive responses to perturbations in cellular homeostasis, *via* modulation of cell metabolism (autophagy response, remodeling of mitochondrial network), participation on danger signaling (such as mitochondrial reactive oxygen species, ROS, or fragments of released mitochondrial DNA) and regulation of programmed cell death (Galluzzi et al., 2012). There are three main reasons pointing mitochondria as the main cellular organelle for CO's biological functions: (i) the main potential candidates for CO to bind are mitochondrial heme-proteins, (ii) CO's biological actions are dependent on mitochondrial ROS signaling and (iii) HO's substrate heme is generated in mitochondrial compartment. Indeed, heme biosynthesis consists of eight sequential enzyme-catalyzed steps. The first and the three last steps of this pathway occur in mitochondria. In the last one, ferrous iron is inserted into protoporphyrin IX by ferrochelatase in mitochondrial matrix (Ajioka et al., 2006).

Therefore, the present mini-review addresses how CO modulates mitochondrial function to promote cell homeostasis and cytoprotection, and to modulate cell metabolism. The main focused processes are: (i) mitochondrial biogenesis, (ii) modulation of enzymatic activity of cytochrome *c* oxidase, (iii) generation of mitochondrial ROS for signaling and (iv) induction of mitochondrial mild uncoupling effect. Furthermore, this review also targets how CO prevents mitochondrial membrane permeabilization (MMP) and consequently programmed cell death.

## CO's binding candidates

Comparing to other gasotransmitters, such as nitric oxide (NO) and hydrogen sulfide (H_2_S), CO is a quite inert molecule. CO needs to be activated by coordination with low-valent metals or ions to chemically react. In biological systems, Fe^2+^ of reduced heme proteins are the main CO targets (Boczkowski et al., 2006; Romão and Vieira, 2013). The best-described candidates are hemoglobin (erythrocytes), myoglobin (myocytes) and cytochrome c oxidase (mitochondrial complex IV).

Cytochrome *c* oxygenase (COX), the final electron acceptor of mitochondrial respiratory chain, was found to be the main mitochondrial target for CO at cytochrome a and a3 (Chance et al., 1970; Brown and Piantadosi, 1990). COX is involved in CO-induced cytotoxicity due to CO capacity of inhibiting its activity and cell respiration (Chance et al., 1970). The binding of CO to COX is highly dependent on oxygen levels, because under hyperbaric oxygen conditions there is dissociation of a cytochrome a and a3-CO complex (Brown and Piantadosi, 1990).

## Cytochrome *c* oxidase (COX) activity and mitochondrial ROS signaling

Although it is largely accepted that CO is cytotoxic by inhibiting COX activity and mitochondrial respiration, low amounts of CO promote cytoprotection *via* modulation of its enzymatic activity. Low amounts of CO induce partial and/or reversible inhibition of COX activity, which accumulates electrons at complex III, generating low amounts of ROS that are important signaling molecules, as reviewed in Bilban et al. (2008), Queiroga et al. (2012). These produced ROS are in low amounts because they are not causing damage, cells still consume oxygen and produce ATP following CO treatment; nevertheless they can be measured by fluorescent dyes (such as MitoSox and DCF) (Vieira et al., 2008; Queiroga et al., 2010, 2011). Furthermore, the CO-reduced inflammation in macrophages is prevented by the addition of N-acetyl-cysteine, which is a precursor of glutathione synthesis, reinforcing the cell anti-oxidant defense. Thus, it indicates that mitochondrial ROS are crucial signaling factors (Zuckerbraun et al., 2007). In mitochondrial DNA-deficient cells, ρ^0^ cells, which are cells deficient in mitochondrial respiration, the anti-inflammatory role of CO is lost (Chin et al., 2007). Likewise, in a model of fulminant hepatitis, overexpression of HO-1 and exogenous CO protect hepatocytes against apoptosis *via* mitochondrial ROS generation, since in ρ^0^ cells no protection and no ROS generation were found (Kim et al., 2008). In airway smooth muscle cells, CORM-2 limits cell proliferation *via* ROS generation due to a decrease on COX activity (Taillé et al., 2005). Furthermore, in isolated liver mitochondria, CO limits MMP and damage, and this effect is reverted by the anti-oxidant β-carotene addition (Queiroga et al., 2011). Nevertheless, one can speculate that CO signaling is not limited to ROS generation, but also involves partial inhibition of mitochondrial respiration. The CO-induced decrease on mitochondrial respiration and ATP production might create a compensatory effect, promoting the activation of mitochondrial biogenesis, which is discussed in the next subsection.

One of the consequences of CO-induced ROS generation is the mitochondrial increase on the ratio between oxidized (GSSG) and reduced (GSH) glutathione, facilitating signaling *via* protein glutathionylation. In non-synaptic mitochondria derived from brain cortex, CO limits mitochondrial damage by promoting glutathionylation of the ATP/ADP carrier in their inner membrane, which improves its function (Queiroga et al., 2010). Furthermore, the cytosolic protein p62 is another example of glutathionylated protein in response to CO. In fact, CORM-2 limits inflammatory responses in endothelial cells *via* mitochondrial ROS generation that promotes glutathionylation of p62, which, in turn, prevents NF-κB activation (Yeh et al., 2014). Finally, in activated macrophages, CO decrease their inflammatory response by partially inhibiting mitochondrial respiration and COX activity, and its effect is exacerbated under hypoxia (D'Amico et al., 2006).

In apparent contrast, there are also data demonstrating that CO improves COX enzymatic activity. In a renal model of diabetes, whose COX activity is lower than control, stimulation of HO-1 expression improves mitochondrial function and COX activity (Di Noia et al., 2006). Stabilization of hypoxia-inducing factor-1α (HIF-1α) improves COX function and optimizes mitochondrial respiration efficiency (Fukuda et al., 2007). Additionally, CO promotes stabilization of HIF-1α in cardiomyocytes (Lakkisto et al., 2010) and macrophages (Chin et al., 2007). Taken all together, one can speculate that (i) CO modulates COX activity *via* stabilization of HIF-1α and/or (II) CO-induced low amounts of ROS promote a compensatory and preconditioning effect that improves mitochondrial function and promotes mitochondrial biogenesis.

One must take into consideration that CO's modulation of COX activity is also dependent on concentration and on period of gas exposure. For instance, *in vivo* exposure of CO gas at 1000 ppm, which are high concentration levels, caused decrease on myocardial COX activity in mice (Iheagwara et al., 2007). While, a two-step response was found in CO-treated mitochondria from liver (Queiroga et al., 2011) and from astrocytes (Almeida et al., 2012). During the first minutes (up to 10) there was a slight decrease on COX activity, while at later times (hours) an increase on specific enzymatic activity was found after CO addition. Thus, a compensatory response of COX activity and oxidative phosphorylation process might be involved following CO's partial inhibition of COX. Likewise, it was previously demonstrated that guinea pig model prolonged exposure to CO resulted in an increase in COX expression in heart and liver, being these responses considered to be adaptive to chronic exposure (Shigezane et al., 1989).

## Mitochondrial biogenesis

Mitochondrial biogenesis seems to be another strategy for the cell to improve metabolism and confer cytoprotection against several damaging stimuli. The axis HO-1/CO positively influences mitochondrial biogenesis in a ROS-signaling dependent pathway, as reviewed in Piantadosi and Suliman (2012). HO-1/CO system reverts the decrease of mitochondrial biogenesis due to doxorubicin-induced cardiotoxicity in a murine model of cardiomyopathy (Suliman et al., 2007a). Also, CORM-3 rescues mice from peritonitis–induced sepsis by supporting cardiac mitochondrial metabolism and decreasing pro-inflammatory biomarkers (TNF-α, NO, and IL-10) in plasma (Lancel et al., 2009). Likewise, CO gas activates HO-1 expression through NF-E2–related factor-2 (Nrf2) transcription factor, promoting mitochondrial biogenesis and rescuing mice from lethal *Staphylococcus aureus* sepsis (MacGarvey et al., 2012). In a human assay, 1 h/day of 250 ppm of CO gas exposure during 5 days increases mitochondrial biogenesis in skeletal muscle cells (Rhodes et al., 2009). Finally, activation of mitochondrial biogenesis is cytoprotective in response to reticulum endoplasmic stress in macrophages (Zheng et al., 2012) and against oxidative stress in astrocytes (Almeida et al., 2012).

Modulation of mitochondrial biogenesis by CO is ROS dependent because: (i) it is controlled by the redox-regulated Nrf2 transcription factor (Piantadosi et al., 2008) and (ii) the anti-oxidant catalase reverts CO-induced mitochondrial biogenesis (Suliman et al., 2007b). In conclusion, mitochondrial biogenesis appears as a compensatory process for pushing cell back to homeostasis, following mitochondrial ROS generation. One can speculate that CO promotes a preconditioning effect and reinforces cellular endogenous cytoprotective mechanisms.

## CO's protection against mitochondrial membrane permeabilization (MMP)

Mitochondrial control of programmed cell death is regulated by many factors presented in the inter-membrane space of mitochondria, in particular inside the cristae compartments. Following cell death activation, factors, such as cytochrome *c*, SMAC or apoptosis-inducing factor (AIF) are released into the cytosol (Kroemer et al., 2007). Thus, MMP is a key event on the release of these pro-cell death factors. As an anti-apoptotic gasotransmitter, CO also modulates MMP. Namely, CO protects PC12 cell (neuronal model) against peroxinitrite-induced cell death, by preventing mitochondrial membrane potential loss (a consequence of MMP) and the release of cytochrome *c* (Li et al., 2006). Hyperoxia-induced lung endothelial cell death occurs *via* intrinsic mitochondrial pathways and CO protects them by inhibiting cytochrome *c* release and activation of caspase 3/9 as well as by preventing Bax translocation into mitochondria and Bid activation, both events which facilitate MMP (Wang et al., 2007). By using isolated mitochondria techniques, it was possible to demonstrate that CO directly targets mitochondria preventing MMP induced by calcium and atractyloside treatment. In non-synaptic mitochondria isolated from brain cortex (Queiroga et al., 2010) and from mitochondria isolated liver (Queiroga et al., 2011), low amounts of CO gas inhibit several processes related to MMP: mitochondrial swelling, loss of mitochondrial membrane potential, permeabilization of inner membrane to molecules lower then 800 Da and the release of cytochrome *c*. Similarly to intact cells, in isolated mitochondria, ROS generation is crucial for CO to regulate MMP, since CO protection is reverted in the presence of β-carotene (Queiroga et al., 2010, 2011).

## Mitochondrial uncoupling and CO

Mitochondrial oxidative metabolism is accompanied by ROS generation due to the incomplete reduction of oxygen into anion superoxide. Under proper control, ROS generation functions as ubiquitous signaling factors. However, under pathological conditions, reversion of electron flow might result in persistent and damaging generation of ROS, thus mild mitochondrial uncoupling is an inherent cellular mechanism to limit oxidative stress. Uncoupling consists of energy dissipation by the leakage of proton through the inner membrane, causing a compensatory increase on oxygen consumption, which is not coupled with ATP production. Iacono and colleagues have demonstrated that CORM-3 protects mitochondria against oxidative stress by inducing mild uncoupling state (Iacono et al., 2011). Low micromolar concentrations of CORM-3 increase oxygen consumption under state 2 of respiration (in an ADP independent manner), indicating an uncoupling effect between oxygen reduction and ATP production. Moreover, an inhibitor of succinate dehydrogenase (complex II), malonate significantly reverses the CORM-3-induced uncoupling effect. Likewise, inhibitors of uncoupling protein (UCP) and ATP/ADP translocator (ANT), which are proteins involved in mitochondrial uncoupling process, also prevented mitochondrial uncoupling due to low concentrations of CORM-3. Whenever respiration is initiated at complex II by using succinate as substrate, there is a reversion of electron transfer to complex I with higher levels of ROS production. Under these conditions, CORM-3 prevented excessive ROS generation, limiting oxidative stress (Iacono et al., 2011). In contrast, when electron transfer is physiologically initiated by addition of pyruvate/malate, CORM-3 promotes ROS generation at complex III due to complex IV inhibition, under a concentration-dependent manner. Since Iacono and colleagues generated these data using an *in vitro* approach (isolated mitochondria), one can speculate whether this CO-promoted mild uncoupling effect is physiologically relevant under pathological conditions.

In a recent and more detailed approach, same authors have found that CO targets phosphate carrier. In fact, by increasing phosphate carrier activity, CO promotes the transport of protons and phosphate inside mitochondria, inducing a mild uncoupling effect (Long et al., 2014).

## Ion channels and CO

Phosphate carrier is not the single channel involved in CO's biological activity. CO also modulates other ion channels. The molecular mechanisms by which this regulation takes place are still uncertain and the direct binding of CO to ion channels is controversial since there is no transition metal present in the structure of these molecules (Peers et al., 2014). Nevertheless, calcium-activated potassium (KCa) channels can bind covalently to heme (Tang et al., 2003), thus one can speculate that CO could indirectly bond through heme group.

CO (CORM-2 and/or dissolved gas) inhibited cardiomyocyte L-type Ca^2+^ currents. This effect was reverted in the presence of mitochondrial complex III inhibitor (antimycin A) or mitochondrial target anti-oxidant (Mito Q), which limits mitochondrial ROS generation (Scragg et al., 2008). Likewise, CO inhibited L-type Ca^2^ channel in a ROS dependent manner, conferring cardioprotection (Dallas et al., 2009). In addition, the loss of intracellular levels of K^+^ is one of the early steps of apoptosis modulation, since intracellular K^+^ maintains mitochondrial potential and volume and cell osmolality. K^+^ efflux occurs *via* potassium channels, and K_v_2.1 channel is particularly involved. In hippocampus CO prevented neuronal apoptosis by selectively inhibiting K_v_2.1 channel. Similarly to Ca^2^ channels, CO inhibits K_v_2.1 channel in a ROS dependent manner, since this effect is reverted in the presence of specific mitochondrial anti-oxidant Mito Q (Dallas et al., 2011).

Finally, calcium transportation into mitochondria is another important and potential CO role, which needs further studies.

## Final remarks

**Figure 1:**
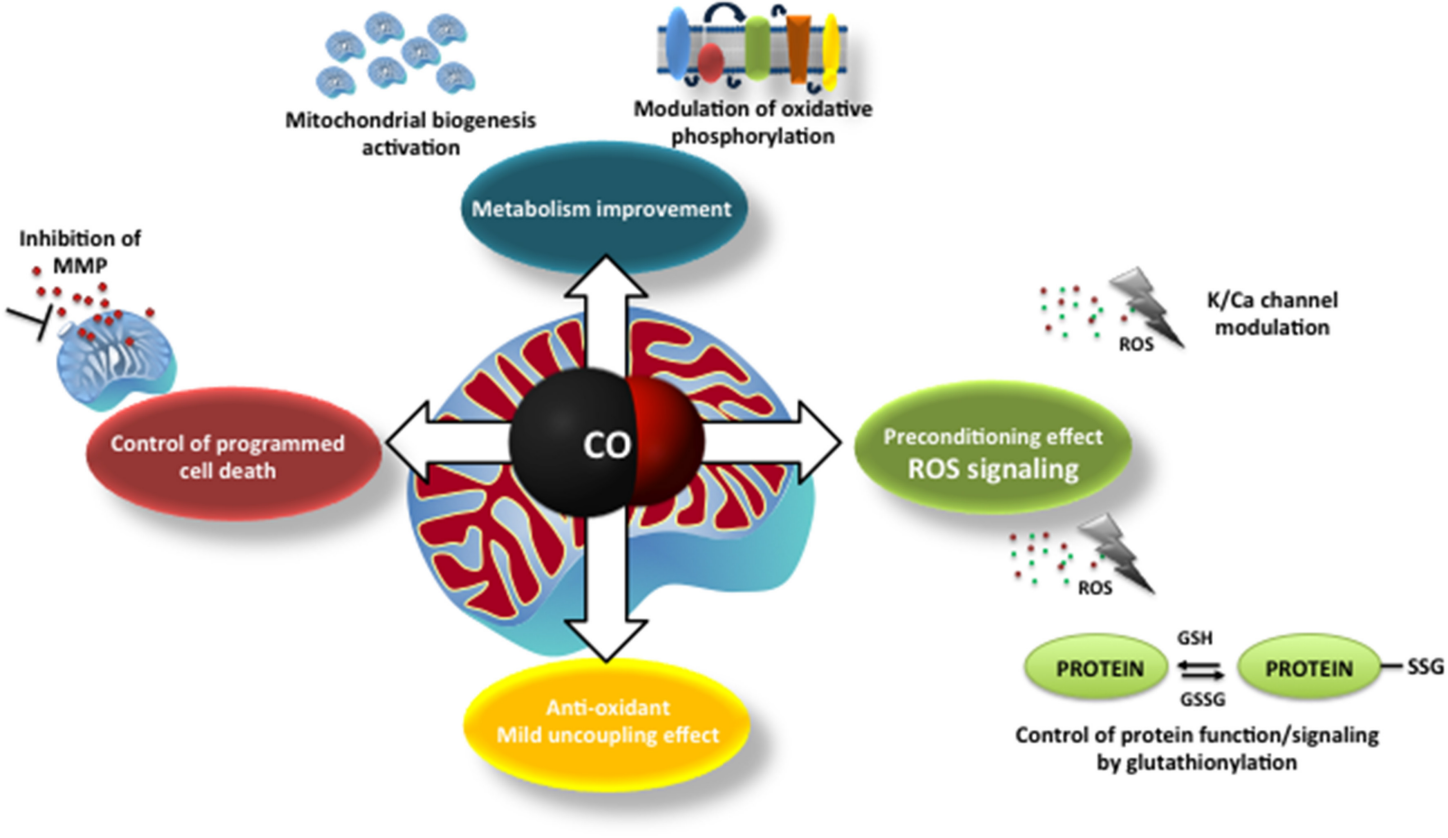
**The main described mechanisms of carbon monoxide on mitochondria: modulation of mitochondrial membrane permeabilization and cell death control; improvement of mitochondrial metabolism (modulation of cytochrome *c* oxidase activity and mitochondrial biogenesis), ROS generation and signaling (redox adaptive cell responses, alert signals) and mild uncoupling effect**.

At first glance, CO mode of action on mitochondria appears to be deleterious, such as: inhibition of COX, ROS generation or uncoupling effect. Nevertheless, the final results are beneficial for the cell, whereas low amounts of ROS generation (i) modulates plasmatic ion channel activity, (ii) improves mitochondrial metabolism, (iii) promotes mitochondrial biogenesis and (iv) prevents MMP and cell death. Likewise, at low concentrations of CO, there is an improvement of COX activity and an induced mild uncoupling that protects mitochondria from oxidative stress. Thus, one can speculate that CO promotes a preconditioning effect at the cellular level. Figure 1 summarizes the main findings concerning CO mode of action.

Nevertheless, several aspects of CO's modulation of mitochondrial function are still unrevealed and faraway from being understood. Namely, CO might affect mitochondrial quality control machinery, probably through ROS signaling. Mitochondrial quality control consists of several processes that must be in balance: (i) mitochondrial adaptation to the cell environment by fusion and fission processes, (ii) elimination of damaged mitochondria by mitochondrial autophagy (mitophagy) and (iii) mitochondrial biogenesis. Further studies are necessary to describe how CO modulates these events and their cross talk.

In addition, mitochondria and endoplasmic reticulum (ER) are physically and functionally related (Naon and Scorrano, 2014). Indeed, ER stress promotes adaptive mitochondrial response (increase on ATP production and mitochondrial biogenesis) through mechanisms that are HO-1/CO dependent (Zheng et al., 2012). However, this inter-organelle communication is still an unexplored field for CO research.

Furthermore, it seems that CO can modulate protein function through redox modulation of critical cysteine residues, following mitochondrial ROS generation. Protein post-translational modifications are key processes for rapid cellular redox response, in particular protein glutathionylation due to the high levels of oxidized glutathione. Thus, it is an aspect of CO's Biology that needs further studies.

Finally, mitochondria emerge as crucial target organelles for the system HO-1/CO, through which cellular homeostasis and survival are modulated.

### Conflict of interest statement

The authors declare that the research was conducted in the absence of any commercial or financial relationships that could be construed as a potential conflict of interest.

## References

[B1] Ajioka R. S. Phillips J. D. Kushner J. P. (2006). Biosynthesis of heme in mammals. Biochim. Biophys. Acta 1763, 723–736. 10.1016/j.bbamcr.2006.05.005 16839620

[B2] Almeida A. S. Queiroga C. S. F. Sousa M. F. Q. Alves P. M. Vieira H. L. A. (2012). Carbon monoxide modulates apoptosis by reinforcing oxidative metabolism in astrocytes: role of Bcl-2. J. Biol. Chem. 287, 10761–10770. 10.1074/jbc.M111.306738 22334654 PMC3322847

[B3] Bilban M. Haschemi A. Wegiel B. Chin B. Y. Wagner O. Otterbein L. E. (2008). Heme oxygenase and carbon monoxide initiate homeostatic signaling. J. Mol. Med. 86, 267–279. 10.1007/s00109-007-0276-0 18034222

[B4] Boczkowski J. Poderoso J. J. Motterlini R. (2006). CO–metal interaction: vital signaling from a lethal gas. Trends Biochem. Sci. 31, 614–621. 10.1016/j.tibs.2006.09.001 16996273

[B5] Brown S. D. Piantadosi C. A. (1990). In vivo binding of carbon monoxide to cytochrome c oxidase in rat brain. J. Appl. Physiol. 68, 604–610. 2156793 10.1152/jappl.1990.68.2.604

[B6] Chance B. Erecinska M. Wagner M. (1970). Mitochondrial responses to carbon monoxide toxicity. Ann. N.Y. Acad. Sci. 174, 193–204. 10.1111/j.1749-6632.1970.tb49786.x 4332407

[B7] Chin B. Y. Jiang G. Wegiel B. Wang H. J. Macdonald T. Zhang X. C.. (2007). Hypoxia-inducible factor 1alpha stabilization by carbon monoxide results in cytoprotective preconditioning. Proc. Natl. Acad. Sci. U.S.A. 104, 5109–5114. 10.1073/pnas.0609611104 17360382 PMC1820823

[B9] Dallas M. L. Boyle J. P. Milligan C. J. Sayer R. Kerrigan T. L. McKinstry C.. (2011). Carbon monoxide protects against oxidant-induced apoptosis via inhibition of Kv2.1. FASEB J. 25, 1519–1530. 10.1096/fj.10-173450 21248240 PMC7615704

[B10] Dallas M. L. Scragg J. L. Peers C. (2009). Inhibition of L-type Ca(2+) channels by carbon monoxide. Adv. Exp. Med. Biol. 648, 89–95. 10.1007/978-90-481-2259-2_10 19536469

[B8] D'Amico G. Lam F. Hagen T. Moncada S. (2006). Inhibition of cellular respiration by endogenously produced carbon monoxide. J. Cell Sci. 119, 2291–2298. 10.1242/jcs.02914 16723735

[B11] Di Noia M. A. Van Driesche S. Palmieri F. Yang L.-M. Quan S. Goodman A. I.. (2006). Heme oxygenase-1 enhances renal mitochondrial transport carriers and cytochrome C oxidase activity in experimental diabetes. J. Biol. Chem. 281, 15687–15693. 10.1074/jbc.M510595200 16595661

[B12] Dore S. Takahashi M. Ferris C. D. Zakhary R. Hester L. D. Guastella D.. (1999). Bilirubin, formed by activation of heme oxygenase-2, protects neurons against oxidative stress injury. Proc. Natl. Acad. Sci. U.S.A. 96, 2445–2450. 10.1073/pnas.96.5.2445 10051662 PMC26804

[B13] Fukuda R. Zhang H. Kim J. W. Shimoda L. Dang C. V. Semenza G. L. (2007). HIF-1 regulates cytochrome oxidase subunits to optimize efficiency of respiration in hypoxic cells. Cell 129, 111–122. 10.1016/j.cell.2007.01.047 17418790

[B14] Galluzzi L. Kepp O. Kroemer G. (2012). Mitochondria: master regulators of danger signalling. Nat. Rev. Mol. Cell Biol. 13, 780–788. 10.1038/nrm3479 23175281

[B15] Gozzelino R. Jeney V. Soares M. P. (2010). Mechanisms of cell protection by heme oxygenase-1. Annu. Rev. Pharmacol. Toxicol. 50, 323–354. 10.1146/annurev.pharmtox.010909.105600 20055707

[B16] Iacono L. L. Boczkowski J. Zini R. Salouage I. Berdeaux A. Motterlini R.. (2011). A carbon monoxide-releasing molecule (CORM-3) uncouples mitochondrial respiration and modulates the production of reactive oxygen species. Free Radic. Biol. Med. 50, 1556–1564. 10.1016/j.freeradbiomed.2011.02.033 21382478

[B17] Iheagwara K. N. Thom S. R. Deutschman C. S. Levy R. J. (2007). Myocardial cytochrome oxidase activity is decreased following carbon monoxide exposure. Biochim. Biophys. Acta 1772, 1112–1116. 10.1016/j.bbadis.2007.06.002 17628447 PMC2045065

[B18] Kim H. S. Loughran P. A. Rao J. Billiar T. R. Zuckerbraun B. S. (2008). Carbon monoxide activates NF-kappaB via ROS generation and Akt pathways to protect against cell death of hepatocytes. Am. J. Physiol. Gastrointest. Liver Physiol. 295, G146–G152. 10.1152/ajpgi.00105.2007 18497334

[B19] Kroemer G. Galluzzi L. Brenner C. (2007). Mitochondrial membrane permeabilization in cell death. Physiol. Rev. 87, 99–163. 10.1152/physrev.00013.2006 17237344

[B20] Lakkisto P. Kyto V. Forsten H. Siren J. M. Segersvard H. Voipio-Pulkki L. M.. (2010). Heme oxygenase-1 and carbon monoxide promote neovascularization after myocardial infarction by modulating the expression of HIF-1alpha, SDF-1alpha and VEGF-B. Eur. J. Pharmacol. 635, 156–164. 10.1016/j.ejphar.2010.02.050 20303947

[B21] Lancel S. Hassoun S. M. Favory R. Decoster B. Motterlini R. Neviere R. (2009). Carbon monoxide rescues mice from lethal sepsis by supporting mitochondrial energetic metabolism and activating mitochondrial biogenesis. J. Pharmacol. Exp. Ther. 329, 641–648. 10.1124/jpet.108.148049 19190234

[B22] Li M. H. Cha Y. N. Surh Y. J. (2006). Carbon monoxide protects PC12 cells from peroxynitrite-induced apoptotic death by preventing the depolarization of mitochondrial transmembrane potential. Biochem. Biophys. Res. Commun. 342, 984–990. 10.1016/j.bbrc.2006.02.046 16598857

[B23] Long R. Salouage I. Berdeaux A. Motterlini R. Morin D. (2014). CORM-3, a water soluble CO-releasing molecule, uncouples mitochondrial respiration via interaction with the phosphate carrier. Biochim. Biophys. Acta 1837, 201–209. 10.1016/j.bbabio.2013.10.002 24161358

[B24] MacGarvey N. C. Suliman H. B. Bartz R. R. Fu P. Withers C. M. Welty-Wolf K. E.. (2012). Activation of mitochondrial biogenesis by heme oxygenase-1-mediated NF-E2-related factor-2 induction rescues mice from lethal Staphylococcus aureus sepsis. Am. J. Respir. Crit. Care Med. 185, 851–861. 10.1164/rccm.201106-1152OC 22312014 PMC3360573

[B25] Motterlini R. Otterbein L. E. (2010). The therapeutic potential of carbon monoxide. Nat. Rev. Drug Discov. 9, 728–743. 10.1038/nrd3228 20811383

[B26] Naon D. Scorrano L. (2014). At the right distance: ER-mitochondria juxtaposition in cell life and death. Biochim. Biophys. Acta 1843, 2184–2194. 10.1016/j.bbamcr.2014.05.011 24875902

[B27] Peers C. Boyle J. P. Scragg J. L. Dallas M. L. Al-Owais M. M. Hettiarachichi N. T.. (2014). Diverse mechanisms underlying the regulation of ion channels by carbon monoxide. Br. J. Pharmacol. [Epub ahead of print]. 10.1111/bph.12760 24818840 PMC4369263

[B28] Piantadosi C. A. Carraway M. S. Babiker A. Suliman H. B. (2008). Heme oxygenase-1 regulates cardiac mitochondrial biogenesis via Nrf2-mediated transcriptional control of nuclear respiratory factor-1. Circ. Res. 103, 1232–1240. 10.1161/01.RES.0000338597.71702.ad 18845810 PMC2694963

[B29] Piantadosi C. A. Suliman H. B. (2012). Redox regulation of mitochondrial biogenesis. Free Radic. Biol. Med. 53, 2043–2053. 10.1016/j.freeradbiomed.2012.09.014 23000245 PMC3604744

[B30] Queiroga C. S. Almeida A. S. Alves P. M. Brenner C. Vieira H. L. (2011). Carbon monoxide prevents hepatic mitochondrial membrane permeabilization. BMC Cell Biol. 12:10. 10.1186/1471-2121-12-10 21388535 PMC3062616

[B31] Queiroga C. S. Almeida A. S. Martel C. Brenner C. Alves P. M. Vieira H. L. (2010). Glutathionylation of adenine nucleotide translocase induced by carbon monoxide prevents mitochondrial membrane permeabilisation and apoptosis. J. Biol. Chem. 285, 17077–17088. 10.1074/jbc.M109.065052 20348099 PMC2878049

[B32] Queiroga C. S. F. Almeida A. S. Vieira H. L. A. (2012). Carbon monoxide targeting mitochondria. Biochem. Res. Int. 2012, 1–9. 10.1155/2012/749845 22536507 PMC3318215

[B33] Queiroga C. S. F. Vercelli A. Vieira H. L. A. (2014). Carbon monoxide and the CNS: challenges and achievements. Br. J. Pharmacol. [Epub ahead of print]. 10.1111/bph.12729 24758548 PMC4369262

[B34] Rhodes M. A. Carraway M. S. Piantadosi C. A. Reynolds C. M. Cherry A. D. Wester T. E.. (2009). Carbon monoxide, skeletal muscle oxidative stress, and mitochondrial biogenesis in humans. Am. J. Physiol. Heart Circ. Physiol. 297, H392–H399. 10.1152/ajpheart.00164.2009 19465554 PMC2711725

[B35] Rodella L. Lamon B. D. Rezzani R. Sangras B. Goodman A. I. Falck J. R.. (2006). Carbon monoxide and biliverdin prevent endothelial cell sloughing in rats with type I diabetes. Free Radic. Biol. Med. 40, 2198–2205. 10.1016/j.freeradbiomed.2006.02.018 16785033

[B36] Romao C. C. Blattler W. A. Seixas J. D. Bernardes G. J. (2012). Developing drug molecules for therapy with carbon monoxide. Chem. Soc. Rev. 41, 3571–3583. 10.1039/c2cs15317c 22349541

[B37] Romão C. C. Vieira H. L. A. (2013). Metal Carbonyls for CO-based Therapies: challenges and successes, in Advances in Organometallic Chemistry and Catalysis ICOMC Silver/Gold Jubilee Book, ed Pombeiro A. J. L. (Wiley-Blackwell), 545–561.

[B38] Ryter S. W. (2006). Heme oxygenase-1/carbon monoxide: from basic science to therapeutic applications. Physiol. Rev. 86, 583–650. 10.1152/physrev.00011.2005 16601269

[B39] Scragg J. L. Dallas M. L. Wilkinson J. A. Varadi G. Peers C. (2008). Carbon monoxide inhibits l-type Ca2+ channels via redox modulation of key cysteine residues by mitochondrial reactive oxygen species. J. Biol. Chem. 283, 24412–24419. 10.1074/jbc.M803037200 18596041 PMC3259849

[B40] Shigezane J. Kita T. Furuya Y. (1989). Acute and chronic effects of carbon monoxide on mitochondrial function. Igaku Kenkyu 59, 35–45. 2560888

[B41] Sjostrand T. (1949). Endogenous formation of carbon monoxide in man. Nature 164, 580. 10.1038/164580a0 18148861

[B42] Suliman H. B. Carraway M. S. Ali A. S. Reynolds C. M. Welty-Wolf K. E. Piantadosi C. A. (2007a). The CO/HO system reverses inhibition of mitochondrial biogenesis and prevents murine doxorubicin cardiomyopathy. J. Clin. Invest. 117, 3730–3741. 10.1172/JCI32967 18037988 PMC2082137

[B43] Suliman H. B. Carraway M. S. Tatro L. G. Piantadosi C. A. (2007b). A new activating role for CO in cardiac mitochondrial biogenesis. J. Cell Sci. 120, 299–308. 10.1242/jcs.03318 17179207

[B44] Taillé C. El-Benna J. Lanone S. Boczkowski J. Motterlini R. (2005). Mitochondrial respiratory chain and NAD(P)H oxidase are targets for the antiproliferative effect of carbon monoxide in human airway smooth muscle. J. Biol. Chem. 280, 25350–25360. 10.1074/jbc.M503512200 15863496

[B45] Tang X. D. Xu R. Reynolds M. F. Garcia M. L. Heinemann S. H. Hoshi T. (2003). Haem can bind to and inhibit mammalian calcium-dependent Slo1 BK channels. Nature 425, 531–535. 10.1038/nature02003 14523450

[B46] Tenhunen R. Marver H. S. Schmid R. (1968). The enzymatic conversion of heme to bilirubin by microsomal heme oxygenase. Proc. Natl. Acad. Sci. U.S.A. 61, 748–755. 10.1073/pnas.61.2.748 4386763 PMC225223

[B47] Vieira H. L. Queiroga C. S. Alves P. M. (2008). Preconditioning induced by carbon monoxide provides neuronal protection against Apoptosis. J. Neurochem. 107, 375–384. 10.1111/j.1471-4159.2008.05610.x 18691384

[B48] Wang X. Wang Y. Kim H. P. Nakahira K. Ryter S. W. Choi A. M. K. (2007). Carbon monoxide protects against hyperoxia-induced endothelial cell apoptosis by inhibiting reactive oxygen species formation. J. Biol. Chem. 282, 1718–1726. 10.1074/jbc.M607610200 17135272

[B49] Yeh P.-Y. Li C.-Y. Hsieh C.-W. Yang Y.-C. Yang P.-M. Wung B. -S. (2014). CO-releasing molecules and increased heme oxygenase-1 induce protein S-glutathionylation to modulate NF-κ B activity in endothelial cells. Free Radic. Biol. Med. 70, 1–13. 10.1016/j.freeradbiomed.2014.01.042 24512908

[B50] Zheng M. Kim S.-K. Joe Y. Back S. H. Cho H. R. Kim H. P.. (2012). Sensing endoplasmic reticulum stress by protein kinase RNA-like endoplasmic reticulum kinase promotes adaptive mitochondrial DNA biogenesis and cell survival via heme oxygenase-1/carbon monoxide activity. FASEB J. 26, 2558–2568. 10.1096/fj.11-199604 22391129

[B51] Zuckerbraun B. S. Chin B. Y. Bilban M. de Costa d'Avila J. Rao J. Billiar T. R.. (2007). Carbon monoxide signals via inhibition of cytochrome c oxidase and generation of mitochondrial reactive oxygen species. FASEB J. 21, 1099–1106. 10.1096/fj.06-6644com 17264172

